# When Fibrosis Mimics Cancer: A Rare Case of Idiopathic Sclerosing Mesenteritis Presenting as Locally Advanced Colonic Neoplasia

**DOI:** 10.1155/cris/5052821

**Published:** 2026-10-30

**Authors:** Adelaide Andriani, Daniele Lomiento, Enrica Avezzù Pignatelli di Montecalvo, Amin Titi

**Affiliations:** ^1^ Department of General Surgery E. Morelli Hospital ASST Valtellina e Alto Lario Sondalo Italy asst-val.it; ^2^ Department of General Surgery Istituto Clinico Sant’Anna Gruppo San Donato Brescia Italy santanna-gsd.it

**Keywords:** abdominal pain, bowel obstruction, case report, computed tomography, differential diagnosis, fibroinflammatory disease, mesenteric mass, mesenteric panniculitis, retractile mesenteritis, sclerosing mesenteritis

## Abstract

Mesenteric panniculitis (MP) is a rare benign fibroinflammatory disorder of mesenteric adipose tissue characterized by variable degrees of fat necrosis, chronic inflammation, and fibrosis. Sclerosing mesenteritis (SM) represents a predominantly fibrotic manifestation within this disease spectrum and may produce a mass‐like, infiltrative appearance that closely mimics malignant intra‐abdominal disease, making preoperative diagnosis particularly challenging. We report the case of a 56‐year‐old man who presented with progressive abdominal pain and signs of subacute intestinal obstruction. Abdominal computed tomography (CT) revealed a stenosing lesion involving the transverse colon with extension to the mesentery and anterior abdominal wall, raising strong suspicion for locally advanced colonic malignancy. The patient subsequently underwent exploratory laparotomy with segmental resection of the transverse colon and en bloc excision of the involved abdominal wall tissue. Histopathological examination unexpectedly demonstrated idiopathic SM with marked fibroinflammatory changes and no evidence of malignancy. The postoperative course was uneventful, and the patient showed progressive clinical improvement during follow‐up. This case highlights the diagnostic difficulty of SM and its ability to clinically and radiologically simulate invasive neoplastic disease. Awareness of this rare entity is essential to guide appropriate management and avoid misdiagnosis.

## 1. Introduction

Mesenteric panniculitis (MP) is a rare, benign fibroinflammatory disorder involving mesenteric adipose tissue, most commonly the small‐bowel mesentery. Histologically, it is characterized by variable combinations of fat necrosis, chronic inflammation, and fibrosis [1]. The terminology surrounding this condition has historically been inconsistent, and the terms mesenteric lipodystrophy, MP, retractile mesenteritis, and sclerosing mesenteritis (SM) have been used to describe different predominant histopathological patterns within a related disease spectrum [2, 3]. Mesenteric lipodystrophy is characterized predominantly by fat necrosis, MP by chronic inflammation, and SM or retractile mesenteritis by prominent fibrosis and tissue retraction [2]. The latter has therefore been proposed to represent a late, predominantly fibrotic manifestation of the spectrum; however, progression from an inflammatory MP phenotype to retractile fibrosis has not been consistently demonstrated, and the nomenclature remains heterogeneous in the literature [1–3].

The reported radiological prevalence of MP varies widely, approximately from 0.16% to 7.8%, depending on patient selection and the imaging criteria used [1, 4]. The condition is most frequently identified in individuals in the fifth to seventh decades of life and generally shows a male predominance [1, 4]. Its etiology remains uncertain, although previous abdominal surgery or trauma, autoimmune conditions, ischemia, infection, and malignancy have all been proposed as associated factors [1, 3]. In patients without an identifiable underlying cause, the disease is considered idiopathic.

The clinical presentation of MP and its sclerosing manifestations is highly variable. Many patients are asymptomatic and are diagnosed incidentally on cross‐sectional imaging, whereas symptomatic patients may present with abdominal pain, bloating, nausea, altered bowel habits, weight loss, or other nonspecific gastrointestinal symptoms [1, 3]. In more advanced fibrotic disease, mass effect and retraction may occasionally result in complications such as bowel obstruction, vascular compromise, or ischemia [3]. Computed tomography (CT) is the principal imaging modality for diagnosis, although histopathological confirmation may be required in atypical or mass‐forming presentations, particularly when malignancy cannot be confidently excluded [3, 5].

Management depends primarily on symptom burden and the presence of complications. Asymptomatic patients generally require observation alone, whereas medical therapy may be considered in symptomatic disease; surgery is usually reserved for complications such as persistent bowel obstruction or for cases in which malignancy cannot be adequately excluded [3, 5]. Because high‐quality prospective therapeutic evidence remains extremely limited, treatment recommendations are largely derived from small observational series, expert guidance, and a few small prospective pilot studies [5]. Here, we describe an unusual case of idiopathic SM with predominant omental and colonic involvement, presenting as an infiltrative pseudotumoral lesion that closely mimicked locally advanced colonic malignancy.

## 2. Case Presentation

In March 2026, a 56‐year‐old Moroccan man, who had been living in Italy for several years, presented to the Emergency Department with persistent diffuse abdominal pain, predominantly localized to the mesogastric region, associated with constipation and clinical features suggestive of subacute intestinal obstruction. The patient was afebrile and denied urinary symptoms, recent travel abroad, ingestion of raw or poorly preserved food, or episodes of vomiting.

On physical examination, the patient appeared clinically distressed. The abdomen was distended and diffusely tender on palpation, with a palpable area of increased consistency in the mesogastric region. Bowel sounds were reduced and hypoactive. Initial laboratory investigations showed leukocytosis (WBC 14 × 10^3^/μL) and elevated C‐reactive protein levels (13.4 mg/dL), while liver function tests were within normal limits. Serum creatinine was mildly elevated (1.25 mg/dL).

Abdominal ultrasonography revealed a complex nodular lesion measuring ~3 cm within the omental region between the mesogastrium and the right flank, associated with a small amount of free fluid in the right paracolic gutter. A contrast‐enhanced abdominal CT scan was subsequently performed for further evaluation. Imaging demonstrated a markedly heterogeneous solid thickening located anterior to the transverse colon in the right paramedian omental region, measuring ~4.3 cm × 12 cm in axial diameter and extending cranio‐caudally for more than 6 cm. The lesion exhibited a denser central component containing punctate calcification and a less dense peripheral portion, with poor enhancement after contrast administration. The mass appeared adherent to the anterior abdominal wall, where focal thickening of the right rectus abdominis muscle was noted, and no clear cleavage plane with the transverse colon could be identified posteriorly. No peritoneal effusion was detected. Mild thickening of the diaphragmatic profile was observed, while the liver, biliary tree, pancreas, spleen, adrenal glands, and kidneys appeared to be unremarkable. No pelvic abnormalities were identified (Figure 1).

**Figure 1:**
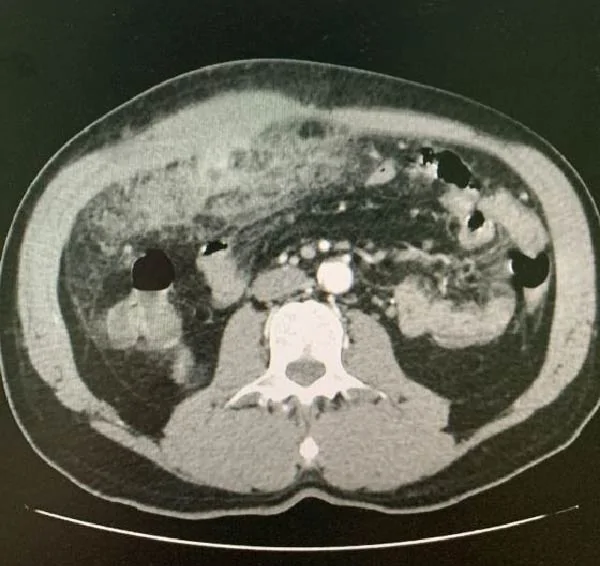
Contrast‐enhanced abdominal CT scan showing a heterogeneous omental mass adherent to the transverse colon and anterior abdominal wall, with central calcification and pseudotumoral appearance.

Given the radiologic appearance of an “omental cake,” a neoplastic process was strongly suspected, and the patient was referred for surgical evaluation and admitted to the General Surgery Unit for further diagnostic workup. Supportive treatment with bowel rest, intravenous fluids, electrolyte replacement, and analgesic therapy was initiated.

Subsequent oncologic markers revealed normal alpha‐fetoprotein, CEA, and CA 19‐9 levels, whereas CA‐125 was mildly elevated (56.5 U/mL). Serological testing for HIV, HBV, and HCV was negative, as were serum IgG4 levels. An ultrasound‐guided core needle biopsy of the omental lesion was then performed. Histopathological examination demonstrated peritoneal tissue fragments infiltrated by dense lymphoplasmacytic inflammatory cells embedded within the fibrotic stroma, with scattered granulocytic and predominantly plasmacytic components arranged both singly and in small aggregates. Immunohistochemistry was negative for cytokeratin AE1/AE3, with only rare CD20+ and CD3+ lymphocytes being identified.

Despite conservative management, the patient experienced progressive worsening of abdominal pain and obstructive symptoms. Ten days after admission, emergency exploratory laparotomy was undertaken. Intraoperatively, a large infiltrative mass involving the anterior abdominal wall, falciform ligament, and transverse colon was identified. En bloc resection was therefore performed, including segmental transverse colectomy, partial omentectomy, and excision of the involved portion of the anterior abdominal wall musculature, followed by termino‐terminal colo‐colic anastomosis and placement of a perianastomotic drain (Figure 2).

**Figure 2:**
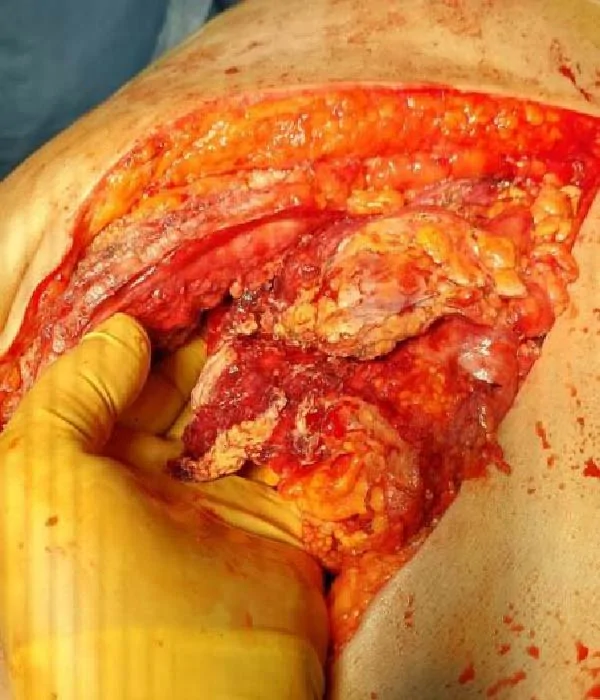
Intraoperative view demonstrating the infiltrative fibroinflammatory mass involving the omentum, transverse colon, and anterior abdominal wall.

The postoperative course was uneventful, with no surgical complications. Definitive histopathological examination revealed a predominantly fibrosing fibroinflammatory process involving the greater omentum and transverse colon, consistent with SM. Microscopic evaluation showed alternating fibrotic and inflammatory areas within an edematous stroma with myxoid degeneration, producing solid pseudoinfiltrative features extending toward the colonic wall. Although SM typically affects the mesentery, less common localizations involving the omentum and colon have been described.

The patient was discharged on postoperative Day 7. At an outpatient follow‐up 15 days later, he was in good general condition, asymptomatic, and with normal bowel function.

## 3. Discussion

MP is an uncommon benign fibroinflammatory disorder of mesenteric adipose tissue with a heterogeneous clinical, radiological, and histopathological presentation [1]. Its terminology has evolved considerably and remains a source of confusion. Histopathologically, fat necrosis, chronic inflammation, and fibrosis may coexist in variable proportions, giving rise to the historically used terms mesenteric lipodystrophy, MP, and sclerosing or retractile mesenteritis [2, 3]. In the classic spectrum model, MP denotes an inflammation‐predominant phenotype, whereas sclerosing or retractile mesenteritis denotes a fibrosis‐predominant phenotype and has been hypothesized to represent a later stage of the process [2]. However, longitudinal evidence demonstrating consistent progression from MP to retractile fibrosis is limited, and some contemporary reviews therefore caution against considering this sequence inevitable [1, 3].

This distinction is particularly relevant to the present case. The marked fibrosis, pseudoinfiltrative growth pattern, adherence to adjacent structures, and extension toward the colonic wall support classification as SM rather than uncomplicated inflammatory MP. The etiology of this spectrum remains incompletely understood. Proposed associations include previous abdominal surgery or trauma, autoimmune dysregulation, ischemia, infection, and malignancy, although many cases remain idiopathic [1, 3].

The clinical manifestations across the MP–SM spectrum are nonspecific and often insidious. Abdominal pain is the most frequently reported symptom, followed by nausea, weight loss, altered bowel habits, abdominal distension, and, less commonly, bowel obstruction or mesenteric ischemia [1, 2, 6]. In our patient, the presentation with persistent abdominal pain and progressive subocclusive symptoms initially suggested an advanced neoplastic process involving the transverse colon. The presence of an infiltrative omental mass with extension to the anterior abdominal wall further reinforced the suspicion of malignancy.

The relationship between MP and malignancy deserves particular consideration because the two conditions may coexist and, as in the present case, may also closely resemble one another radiologically. In a cohort of 359 patients with CT‐reported mesenteric abnormalities consistent with panniculitis, Smith et al. [6] found that 22.6% had a known malignancy at the index examination, whereas 6.8% of patients without a previously known malignancy were diagnosed with cancer during evaluation of the index CT findings. However, whether MP itself confers an increased risk of malignancy remains controversial. A systematic review by Halligan et al. [7] found substantial heterogeneity and methodological bias among available imaging studies and concluded that a causal or predictive association with subsequent malignancy could not be established with certainty. More recently, a meta‐analysis of matched controlled studies including 415 patients with MP and 1132 controls found no significant increase in the odds of malignancy (pooled OR 0.91, 95% CI 0.69–1.20) [8]. These findings suggest that MP should not be regarded as an established paraneoplastic condition; nevertheless, atypical imaging features, progressive symptoms, or a mass‐forming infiltrative pattern should prompt careful exclusion of an underlying malignancy [5–8].

Radiologically, MP and its sclerosing manifestations may present with a broad spectrum of findings, ranging from increased mesenteric fat attenuation or “misty mesentery” to a mass‐like fibrotic process with a pseudotumoral appearance [1, 3]. The CT findings observed in our case, namely, the heterogeneous omental thickening, punctate calcification, absence of a clear cleavage plane with the transverse colon, and infiltration of the abdominal wall, were highly concerning for peritoneal carcinomatosis or locally advanced colonic neoplasia. Similar radiological features have been described in previous reports in which SM simulated mesenteric tumors, carcinomatosis, lymphoma, or invasive gastrointestinal malignancies [3, 4, 7]. This overlap frequently makes a definitive preoperative diagnosis difficult.

Although CT is central to the diagnosis of MP/SM, histopathological confirmation remains important in atypical or mass‐forming presentations when malignancy cannot be confidently excluded [3, 5]. In the present case, ultrasound‐guided biopsy demonstrated a dense lymphoplasmacytic inflammatory infiltrate embedded in the fibrotic stroma, without evidence of epithelial neoplasia. Nevertheless, because of the patient’s progressive clinical deterioration and persistent suspicion of an invasive disease, surgical exploration became unavoidable.

The intraoperative findings of a firm infiltrative mass involving the omentum, transverse colon, falciform ligament, and anterior abdominal wall represented an unusual localization and pattern of extension. MP and SM classically involve the small‐bowel mesentery, whereas predominant omental and colonic involvement is considerably less common The marked fibroinflammatory reaction observed histologically explained the pseudoinfiltrative appearance of the lesion and its close adherence to surrounding structures. This aggressive macroscopic presentation significantly contributed to the diagnostic dilemma.

Therapeutic evidence for MP and SM remains limited, and treatment should be individualized according to symptom severity and the presence of complications. Asymptomatic patients generally require no specific therapy [1, 5]. In symptomatic disease, corticosteroids and other anti‐inflammatory or immunomodulatory agents have been used, although robust prospective evidence is scarce. Two small prospective open‐label studies have specifically evaluated treatment in symptomatic MP using the Mesenteric Panniculitis Subjective Assessment Score (MPSAS). Ginsburg and Ehrenpreis treated five patients with thalidomide 200 mg nightly for 12 weeks; four of five experienced overall symptomatic improvement, accompanied by reductions in inflammatory markers [9]. A subsequent open‐label study evaluated low‐dose naltrexone in three patients with symptomatic MP and reported improvement in MPSAS, although the extremely small sample size precludes firm conclusions [10].

Tamoxifen, usually combined with prednisone, has also been widely cited as a treatment option for symptomatic sclerosing disease. However, this recommendation is based predominantly on observational rather than randomized prospective evidence. In the Mayo Clinic series reported by Akram et al. [11], 20 patients received tamoxifen in combination with a prednisone taper, of whom 12 (60%) improved within 12–16 weeks. No randomized controlled trial has established the efficacy of this regimen [3, 5]. Tamoxifen therapy also requires careful assessment of its potential adverse effects. Common adverse effects include hot flashes and other endocrine symptoms, whereas clinically important complications include venous thromboembolism and, particularly with prolonged exposure, an increased risk of endometrial pathology; thromboembolic events were reported in 3 of 19 tamoxifen‐treated patients in the Mayo Clinic series [11, 12]. Thus, the potential benefits of tamoxifen should be balanced against individual thromboembolic and other risk factors.

Surgical intervention is generally reserved for complications such as persistent bowel obstruction, ischemia, perforation, or situations in which malignancy cannot be adequately excluded [3, 5, 11]. In the present case, progressive obstructive symptoms despite conservative treatment, together with the highly infiltrative radiological and intraoperative appearance, made surgical exploration and resection necessary.

This case highlights several clinically relevant aspects of SM. First, the disease may present as an aggressive pseudotumoral lesion with involvement extending beyond the mesentery, closely resembling advanced malignancy. Second, despite advances in imaging and minimally invasive biopsy techniques, definitive diagnosis may still require surgical resection in selected patients. Finally, awareness of this rare entity is essential for both surgeons and radiologists in order to include it in the differential diagnosis of omental and mesenteric masses, particularly in patients presenting with subocclusive symptoms and inconclusive histological findings.

The present case further emphasizes how fibroinflammatory diseases of the mesentery may closely reproduce the biological and radiological behavior of infiltrative malignancies, representing a significant diagnostic pitfall even in tertiary referral centers.

## 4. Conclusions

Idiopathic SM is a rare and diagnostically challenging fibroinflammatory disease that may clinically and radiologically mimic advanced intra‐abdominal malignancy. The nonspecific nature of its presentation, together with the absence of pathognomonic laboratory or imaging findings, often makes preoperative diagnosis difficult and may lead to surgical exploration, particularly in patients presenting with obstructive symptoms or infiltrative abdominal masses.

The present case highlights an uncommon presentation with predominant omental and colonic involvement associated with extension to the anterior abdominal wall, initially raising strong suspicion for locally advanced transverse colon cancer. Despite modern imaging techniques and percutaneous biopsy, a definitive diagnosis was achieved only after surgical resection and histopathological examination.

Although SM generally follows a benign clinical course, awareness of this entity is essential in order to include it in the differential diagnosis of mesenteric and omental masses. Early recognition may help avoid unnecessary aggressive procedures and allow for a more tailored therapeutic approach. Further studies are needed to better define the pathogenesis, diagnostic criteria, and optimal management of this uncommon disease.

## Funding

No external funding was received for this study.

## Ethics Statement

Ethics Committee approval was not required for this single‐patient case report as it describes routine clinical care without any experimental intervention, in accordance with applicable institutional policies.

## Consent

Written informed consent was obtained from the patient for publication of this case report and accompanying images.

## Conflicts of Interest

The authors declare no conflicts of interest.

## Data Availability

The data supporting the findings of this case report are included in the article. Additional de‐identified information may be made available from the corresponding author upon reasonable request, subject to patient confidentiality and applicable institutional policies.
